# Effectiveness of Eye Movement Desensitization and Reprocessing (EMDR) in Treating Borderline Personality Disorder: A Randomized Controlled Trial

**DOI:** 10.31083/AP40031

**Published:** 2025-04-28

**Authors:** Marcelo Nvo-Fernandez, Fabiola Salas, Valentina Miño-Reyes, Francisco Ahumada-Méndez, Pablo Medina, Daniela Avello, Síbila Floriano Landim, Marc Via, Nicholas Napolitano, Marcelo Leiva-Bianchi

**Affiliations:** ^1^Laboratory of Methodology for Behavioral Sciences and Neurosciences, Faculty of Psychology, Universidad de Talca, 3460000 Talca, Chile; ^2^Departamento de Terapia Ocupacional, Escuela de Ciencias de la Salud, Facultad de Medicina. Pontificia Universidad Católica de Chile, 8940000 Santiago, Chile; ^3^Centro de Desarrollo de Tecnologías de Inclusión (CEDETI UC), Pontificia Universidad Católica de Chile, 8940000 Santiago, Chile; ^4^School of Occupational Therapy, Faculty of Psychology, Universidad de Talca, 3465548 Talca, Chile; ^5^Brainlab-Grup de Recerca en Neurociència Cognitiva, Departament de Psicologia Clínica i Psicobiologia, Institut de Neurociències, Universitat de Barcelona, 08035 Barcelona, Spain; ^6^Cognitive Neuroscience Group, Institut de Recerca Sant Joan de Déu, 08950 Esplugues de Llobregat, Spain; ^7^Faculty of Psychology, Universidad de Talca, 3460000 Talca, Chile

**Keywords:** eye movement desensitization and reprocessing, borderline personality disorder, randomized controlled trial, post-traumatic stress disorder, complex post-traumatic stress disorder, post-traumatic growth

## Abstract

**Background:**

Eye movement desensitization and reprocessing (EMDR) is recommended by major health organizations for trauma treatment, but its efficacy for borderline personality disorder (BPD) remains unestablished. This study aims to evaluate EMDR’s effectiveness in treating BPD through a randomized controlled trial (RCT) and compare its outcomes with cognitive behavioral therapy (CBT).

**Methods:**

A total of 76 individuals participated in the RCT, with 18 patients (78% female) completing the study. Participants were randomly assigned to receive either EMDR (n = 8) or CBT (n = 10) via teletherapy sessions. Trauma symptoms were assessed using the international trauma questionnaire (ITQ), BPD symptoms were assessed using the Personality Assessment Inventory-Borderline Features Scale (PAI-BOR), and post-traumatic growth (PTG) was assessed using the post-traumatic growth inventory (PTGI). Additionally, attentional evaluations were conducted at behavioral and electroencephalographic levels through an oddball paradigm. A final comparison was made between a participant who did not complete the therapeutic process and a participant who did.

**Results:**

Both EMDR and CBT treatments significantly improved trauma and BPD symptoms, as well as post-traumatic growth. The effect size was moderate for ITQ (η^2^ = 0.615) and PTGI (η^2^ = 0.610), and low for PAI-BOR (η^2^ = 0.147). Symptomatic participants showed a decrease in ITQ (*p* = 0.006) and PAI-BOR (*p* = 0.047) scores, and an increase in PTGI scores (*p* = 0.028).

**Conclusions:**

Both EMDR and CBT significantly improved trauma and BPD symptoms, as well as post-traumatic growth. Additionally, EMDR showed benefits in response accuracy and speed, with a correct response rate of 97% when comparing two participants (with and without therapy). However, completely clean electroencephalography (EEG) data were not obtained from both participants for a deeper comparison.

**Clinical Trial Registration:**

The study was registered at https://doi.org/10.1186/ISRCTN91146045, registration number: ISRCTN91146045, registration date: 21 May 2021.

## Main Points

1. Effectiveness of Eye Movement Desensitization and Reprocessing for borderline personality disorder: This study shows that eye movement desensitization and reprocessing (EMDR) is effective in treating borderline personality disorder (BPD), with outcomes comparable to cognitive behavioral therapy (CBT).

2. Reduction of Trauma-Related Symptoms: Both EMDR and CBT significantly reduce symptoms of post-traumatic stress disorder (PTSD) and complex PTSD (CPTSD), demonstrating their effectiveness in managing trauma.

3. Promotion of Post-Traumatic Growth: Participants receiving EMDR showed significant improvements in post-traumatic growth, indicating that EMDR not only alleviates negative symptoms but also fosters positive psychological development.

4. Behavioral evidence: Evaluations using an oddball paradigm revealed that EMDR improves response accuracy and speed. Although the electroencephalography (EEG) data were not completely clean, these behavioral results support the effectiveness of EMDR from a behavioral perspective.

5. Feasibility of Teletherapy: The study supports the use of EMDR via teletherapy, ensuring high treatment adherence and patient satisfaction, making the therapy accessible to a wider population.

## 1. Introduction

Eye Movement Desensitization and Reprocessing (EMDR) is a treatment for trauma that is as effective as cognitive-behavioral therapy (CBT) [1, 2, 3, 4, 5] and has been recommended by the American Psychological Association [6] as well as the World Health Organization [7]. However, a recent meta-analytic finding from randomized controlled trials (RCTs) suggests that its effect might be low [8], in contrast to the higher effect reported by other meta-analyses [9, 10, 11]. This is also at odds with the evidence supporting EMDR as an effective treatment for other mental health conditions comorbid with trauma, such as psychosis, bipolar disorder, depression, anxiety, addictions, chronic pain, and externalizing behaviors [12, 13]. This controversy reinforces the need for continued research into the processes that explain the effectiveness of EMDR for trauma treatment [13].

EMDR therapy has traditionally been applied to people with Post Traumatic Stress Disorder (PTSD). However, the relevance of studying the efficacy of EMDR therapy for Borderline Personality Disorder (BPD) [14] has also been highlighted, as the symptomatology of BPD includes childhood trauma as the main risk factor [15, 16]. In this context, the work of Marylene Cloitre and colleagues [17] is noteworthy, focusing on the three conditions most related to trauma: PTSD, Complex PTSD (CPTSD), and BPD. Using latent class analysis, they have found that PTSD, CPTSD, and BPD share trauma exposure and emotional dysregulation but differ in self-concept stability, relational issues, and impulsivity [13, 17]. In this context, it has been proposed that EMDR therapy is effective for both PTSD and BPD symptomatology because working on the reprocessing of memories associated with the symptomatology is beneficial for both psychopathologies [18]. With respect to PTSD and BPD, the scarcity of RCTs [19] and case studies [18, 20] does not allow for a conclusive response.

This study provides evidence of the previous controversies from a comprehensive perspective, regarding the effectiveness of EMDR in patients with trauma symptoms, particularly BPD. This perspective requires evaluating both disruptive responses (e.g., PTSD-CPTSD) and healthy ones. In fact, theories such as trauma-resilience [7] and psychosocial impact [21] suggest that most individuals exposed to trauma will respond healthily, for instance, with post-traumatic growth (PTG). In this regard, there is controversy about whether psychotherapy is effective in enhancing PTG [4] and this study provides results on this matter. Additionally, applying methods such as electroencephalography (EEG) to evaluate the effectiveness of EMDR broadens this comprehensive perspective on trauma by assessing neurophysiological changes during reprocessing. Unfortunately, this has been scarcely reported [22, 23] leading to generalized explanations [24]. Moreover, recent meta-analyses highlight the need to explain why EEG would be effective in neurofeedback therapies for PTSD, CPTSD, and BPD [25, 26, 27, 28]. Our study extends this explanation by analyzing the behavioral responses (response times and error rates) of two participants using a widely recognized oddball paradigm for assessing cognitive processes such as attention. Furthermore, following adaptations proposed by Sağaltıcı and colleagues [29] in Alpha Psychiatry, our randomized controlled trial evaluates the effect of EMDR in a teletherapy format. This study was conducted in a clinically underexplored population using this method (Latin America, Chile).

## 2. Methods

### 2.1 Participants, Procedure, and Ethical Approval

**Fig. 1.**
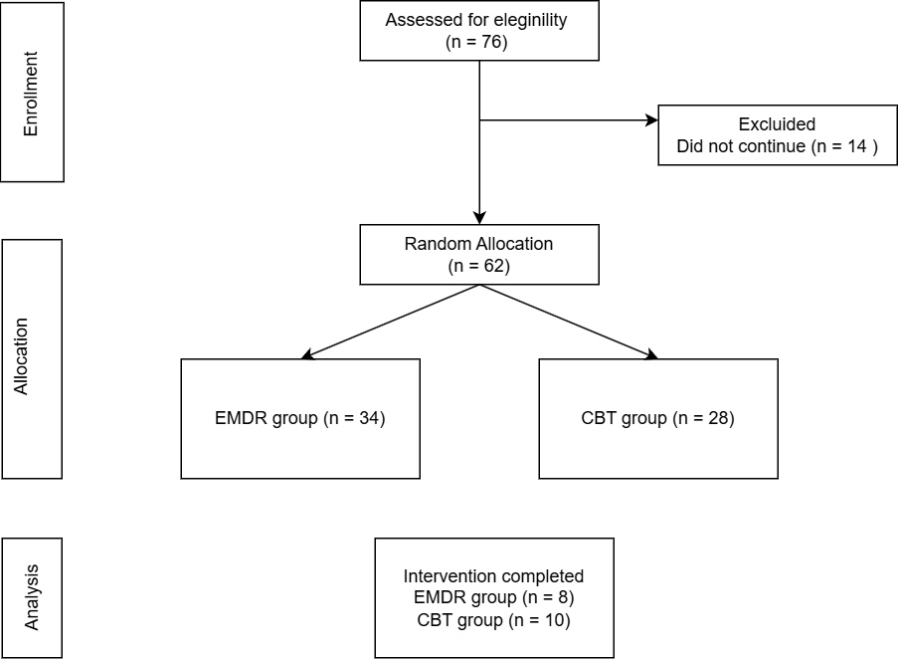
**Participant allocation diagram**.

A power analysis was conducted a priori for two groups and three measurements each, assuming a 20% effect size, a quasi-compliance with sphericity (ε = 0.7), as well as an intermediate inter-measurement correlation (*r* = 0.5). Based on these parameters, the required sample size is 66 participants [30, 31]. Therefore, 76 individuals were enrolled in the RCT and 18 patients (78% female) completed the randomly assigned EMDR (*n* = 8) or CBT (*n* = 10) treatment (24% adherence) using computerized randomization to ensure fairness (see Fig. 1). Among those who adhered, 13 exhibited trauma symptoms (77% female), forming the symptomatic group.

Following the guidelines of the accredited ethics committee that approved this study (registered under ISRCTN91146045; https://doi.org/10.1186/ISRCTN91146045), voluntary participants were included regardless of whether they exhibited trauma symptoms. This strategy allowed for the formation of an asymptomatic group (*n* = 5) consisting of five participants with low symptoms, which helped to control the effects of both treatments. However, the small size of this subgroup limits our ability to adequately control for potential confounding effects (see limitations).

To further explore the neurophysiological explanation for these effects, a case study was conducted. One participant from the symptomatic group who was compliant with the RCT and one from the asymptomatic group who was not compliant with the RCT were evaluated using an oddball paradigm for behavioral and EEG recordings before and after the treatment. Because only two cases were available, this behavioural comparison is an exploratory pilot illustration, and the results are purely descriptive and cannot support inferential conclusions.

Psychotherapy sessions and evaluations were provided free of charge, thanks to funding from the FONDECYT-Chile Project No. 1190578. In order to be eligible for this study, the following criteria were applied: (1) age between 18 and 59 years, a range necessary to standardize EEG procedures [32] and (2) exposure to at least one traumatic event. Participants were excluded if they met any of the following conditions: (1) possible drug or alcohol use disorder, whether mild, moderate, or severe; (2) possible schizophrenia or psychosis; (3) possible bipolar disorder; (4) decompensated personality disorder; (5) possible autism spectrum disorder; (6) high dissociative symptoms; and (7) recent suicide attempt (within the last 6 months). In cases where a role conflict between therapist and patient arose (e.g., student, colleague, family member), a therapist without a conflict of interest was assigned.

### 2.2 Randomization

Participants were randomly assigned to either the EMDR group or the Control group using computerized randomization. The randomization process involved assigning participants to treatment groups in a manner that ensured fairness and minimized bias. Specifically, computer-generated random numbers were used to allocate participants to the treatment groups.

### 2.3 Stratification

Randomization was conducted across the entire participant pool without any stratification by demographic characteristics or any specific criteria. This approach aimed to ensure that the treatment groups were comparable and that any observed differences in outcomes could be attributed to the treatment itself rather than other factors.

### 2.4 Intervention and Outcome Measures

Therapies were administered from August, 2021 to April, 2024 by three therapists trained in EMDR and CBT, all belonging to the Applied Psychology Center at the University of Talca. Due to health and safety concerns related to COVID-19, the teletherapy model was used [29]. Sessions were completely private between therapist and patient, conducted entirely online and encrypted end-to-end using the Zoom platform. The blinding was single-blind; each patient was unaware of the type of therapy they received. However, their participation was fully informed and consented through a document approved by the ethics committee [33]. This document contained general information about both treatments, as well as potential benefits and effects. The team of therapists met weekly for general supervision, as well as monthly to resolve clinical cases with an accredited supervisor.

CBT was structured in stages of screening, framing, assessment, goal construction, treatment, and evaluation. For EMDR therapy, the standard eight-phase protocol was used: (1) patient history, (2) patient preparation, (3) assessment of primary aspects of the traumatic event, (4) desensitization of the traumatic memory, (5) installation of positive cognition, (6) body scan, (7) closure, and (8) reassessment.

Participants were assessed by their therapist at three moments in time: at the beginning (Baseline), middle (Midpoint), and end (Endpoint) of treatment. PTSD symptoms were measured using the International Trauma Questionnaire (ITQ) [34, 35, 36, 37] and BPD symptoms were assessed with the Personality Assessment Inventory-Borderline Features Scale (PAI-BOR) [38, 39, 40]. PTG was measured using the Post-traumatic Growth Inventory (PTGI) [41].

### 2.5 Behavioral and Electroencephalography Measures

The present study used the 64-channel Biosemi ActiveTwo system (BioSemi B.V., Amsterdam, Netherlands) employing the 10/20 positioning system. The flat electrodes, used for EOG and EEG references, were attached to the skin with adhesive rings and electrolyte gel.

A classical auditory oddball paradigm was used for stimulus presentation. The auditory stimuli for the ERP experiment consisted of computer-generated (MATLAB version 2024) sinusoidal tones of 500 Hz (standard tones) and 1000 Hz (target tones), with a duration of 10 ms and a rise and fall time of 5 ms. The paradigm consisted of presenting standard tones at a frequency of 80% and infrequent target tones at a frequency of 20%. The task comprised a fixed pseudo-random sequence of 600 stimuli, with an inter-stimulus interval (ISI) randomly ranging from 200 to 1400 ms, divided into four blocks. The stimuli were delivered at approximately 70 dB through headphones. Participants were shown instructions on a computer, indicating that they should press a response key using their preferred hand to respond to both tones.

It should be noted that prior to the behavioral-EEG recording, each participant underwent an audiometry test following the recommended parameters of 80 dB HL or less, with both participants obtaining normal ranges in the test.

### 2.6 Statistical Analysis

The data were analyzed using JAMOVI 2.2.5 (The JAMOVI Project, Sydney, NSW, Australia) [42] and MATLAB 2023 [43] software (The MathWorks Inc., Natick, MA, USA). For the RCT, the split-plot repeated measures analysis of variance (ANOVA) method was applied. Although this method is standard for RCTs, it presents a challenge as Fisher’s F test may retain or reject the null hypothesis more often than conventionally established for a contrast (α < 0.05; 1-β > 0.95), especially when its assumptions are not met. To address this, the Improved General Approximation (IGA) of Fisher’s F [44, 45, 46] was applied. This method eliminates the necessity of evaluating the assumptions. However, supplementary analyses included: (1) equality of variance among participant groups (homogeneity: Levene’s L; *p* > 0.05); (2) equality of variances between differences in variance for each group across measurement points (sphericity: Mauchly’s W; *p* > 0.05; Greenhouse-Geisser’s ε > 0.75); and (3) normality of distribution within each group and time point (Shapiro-Wilk’s W; *p* > 0.05).

Given that assumption violations are more of the rule rather than the exception in the behavioral sciences [47], using IGA allows for controlling Type I errors, therefore reducing the likelihood of false positives. Additionally, the effect size was evaluated using Generalized η^2^ and classified according to Ferguson’s rule [48] due to its robustness in excluding the variance of other factors in repeated measures models [49, 50]. The means of ITQ, PAI-BOR, and PTGI for each participant group at each measurement point were compared using the Student’s *t*-test (*p* < 0.05), alongside mean and error plots (confidence interval (CI) = 95%).

### 2.7 Preprocessing of EEG Measures

The preprocessing of EEG measurement data was carried out in several critical stages. First, the EEGLAB graphical interface (https://eeglab.org/) was used for an initial visual inspection of the raw EEG data, allowing for the identification and manual marking of segments containing obvious artifacts, such as ocular and muscle movements. Subsequently, a frequency filter was applied using ERPLAB tools, setting a high-pass filter at 0.1 Hz to eliminate slow drifts and a low-pass filter at 30 Hz to remove high-frequency noise, thereby reducing unwanted frequencies that could contaminate the EEG data.

Next, an event list was created using the EventList function in ERPLAB (ERPLAB Toolbox, Center for Mind and Brain, University of California, Davis, CA, USA; https://erpinfo.org/erplab), which is essential for organizing data into different experimental conditions. Events were assigned to specific bins using an appropriate configuration file in ERPLAB, allowing for the structured organization of the various experimental conditions. Subsequently, epochs based on the defined bins were extracted using a –200 to 600 ms window in the BINEPOCHS Graphical User Interface (GUI), thus segmenting the EEG data into more manageable fragments based on the events of interest.

For artifact detection and correction, several methods were implemented. Using the automatic artifact detection function in ERPLAB, epochs containing artifacts were automatically identified based on criteria such as excessive amplitudes, high standard deviations, and abrupt gradients in the EEG signals. Specific thresholds were established for signal amplitude and variability, marking epochs that exceeded these thresholds as artifact-contaminated and excluding them from subsequent analysis. Additionally, regression techniques and independent component analysis (ICA) were employed to identify and eliminate components related to blinks and eye movements, decomposing the EEG signals into independent components to remove those corresponding to ocular artifacts without affecting the neural signals of interest. In cases where artifacts were detected in specific channels, those channels were interpolated using data from neighboring channels to minimize information loss.

After completing the initial stages of EEG data preprocessing, efforts were made to proceed with the analysis of event-related potentials (ERPs). However, a critical limitation emerged due to the substantial presence of noise within the signals, primarily generated by involuntary muscular movements of the participants during data acquisition. This noise, predominantly of electromyographic (EMG) origin, significantly compromised the quality of the EEG signals.

Despite the application of frequency filters, artifact detection, and correction methods, the level of EMG interference persisted, undermining the validity of the data. Muscular artifacts, characterized by their high frequency and amplitude, exceeded the thresholds established for automatic artifact detection, resulting in the exclusion of numerous epochs. This led to a reduced and potentially non-representative dataset, thereby limiting its utility for subsequent analyses.

Attempts at correction through ICA were also unsuccessful in effectively separating neural signals from muscular artifacts. The complexity and non-linear nature of the EMG noise hindered the precise identification of relevant components, thereby negatively impacting the quality of the remaining data. Channel interpolation, a technique employed to mitigate data loss in cases of excessive noise, was likewise insufficient to restore the integrity of the EEG signals in areas most affected by the noise.

Consequently, the preprocessed dataset was deemed inadequate for reliable ERP analysis, as the neural signals of interest were significantly masked by residual noise. This issue underscores the need for more rigorous artifact control strategies during EEG data collection, such as improving participant stabilization or employing advanced noise elimination techniques, to better preserve the cerebral signals of interest for future studies [51, 52].

The methodology adhered to the Consolidated Standards of Reporting Trials (CONSORT) checklist for clinical trials, ensuring transparency and completeness in reporting (see **Supplementary Material**).

## 3. Results

**Fig. 2.**
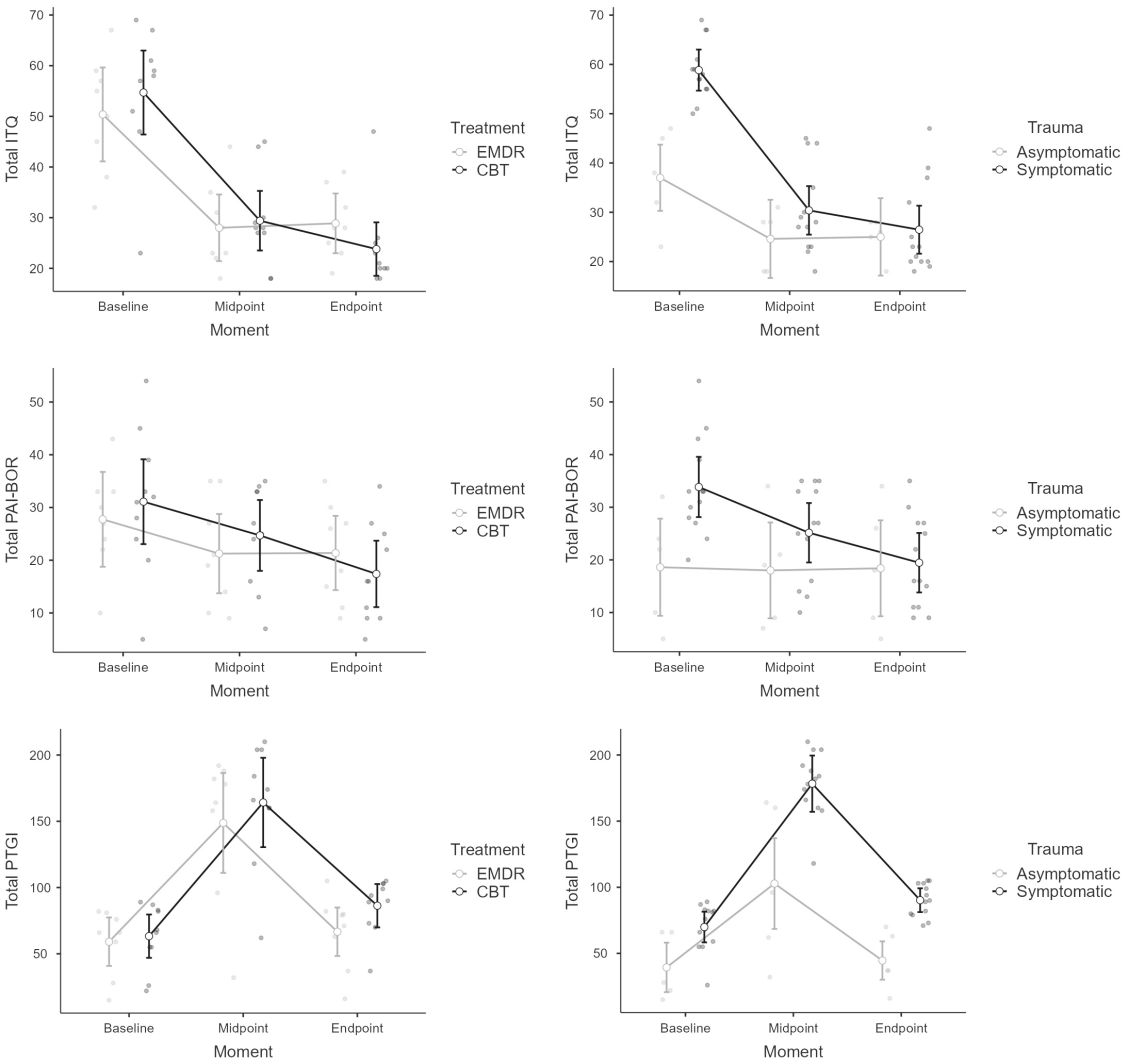
**Mean and error plots of randomized controlled trial (RCT) interactions**. EMDR, eye movement desensitization and reprocessing.

The results indicate a significant effect of both treatments equally. The effect is moderate on ITQ (*Generalized η^2^* = 0.615) and PTGI (*Generalized η^2^* = 0.610), and low on PAI-BOR (*Generalized η^2^* = 0.147; see Table 1, Rows “Moment”). Additionally, there was a slightly significant effect in the symptomatic group. These participants showed a decrease in their total ITQ *(IGA* = 7.33; *p* = 0.006; *Generalized η^2^* = 0.216) and PAI-BOR scores (*IGA* = 4.12; *p* = 0.047; *Generalized η^2^* = 0.129), and an increase in PTGI scores (*IGA* = 6.153; *p* = 0.028; *Generalized η^2^* = 0.109), compared with those without trauma symptoms (see Table 1, Rows “Trauma*Moment”). The comparisons using the Student’s *t*-test reinforce the graphical observations (see Fig. 2). Both the total ITQ and PAI-BOR scores decreased as the treatment progressed, with a significantly higher average in the symptomatic group only at baseline. Conversely, the total PTGI exhibits an inverted V-shaped pattern, being significantly higher at the midpoint measurement among those in the asymptomatic group (see Fig. 2, Table 2).

**Table 1.** **Effect of the Moment factor and interactions (Treatment vs Moment and Trauma vs Moment) in the RCT**.

Total		Fisher’s F	IGA	1-β	Generalized η^2^	Mauchly’s W	Greenhouse-Geisser’s ε
Moment							
ITQ		**50.91; <0.001**	50.91; 1.9324 × 10^-6^	1.000	0.615	–	–
PAI-BOR		**9.85; <0.001**	9.85; 0.008	0.962	0.147	–	–
PTGI		**116.3; <0.001**	116.3; 5.8641 × 10^-5^	1.000	0.610	–	–
Treatment* Moment						–	–
	ITQ	1.35; 0.275	1.35; 0.273	0.269	0.042	**0.413; 0.001**	0.630
	PAI-BOR	1.59; 0.220	1.59; 0.227	0.311	0.002	**0.572; 0.015**	0.700
	PTGI	0.699; 0.504	0.699; 0.378	0.158	0.009	**0.434; 0.002**	0.639
Trauma* Moment							
	ITQ	**7.33; 0.002**	7.33; 0.006	0.915	0.216	**0.614; 0.026**	0.721
	PAI-BOR	**4.12; 0.026**	4.12; 0.047	0.688	0.129	0.695; 0.065	0.766
	PTGI	**6.153; 0.005**	6.153; 0.028	0.859	0.109	**0.654; 0.041**	0.743

Note: Statistic and probability in **bold** when there is a significant difference. RCT, randomized controlled trial; ITQ, International Trauma Questionnaire; PAI-BOR, Personality Assessment Inventory-Borderline Features Scale; IGA, Improved General Approximation; PTGI, Post-Traumatic Growth Inventory, * = interaction between variables.

**Table 2.** **Comparison of means, variances, and distribution of groups at each moment in the RCT**.

		Baseline	Midpoint	Endpoint
Treatment - ITQ				
	Student’s T	–0.738; 0.471	–0.336; 0.741	1.360; 0.193
	Levene’s L	2.227; 0.155	0.637; 0.437	4.165; 0.058
	Shapiro-Wilk’s W	0.984; 0.660	**0.837; <0.001**	**0.836; 0.005**
Trauma - ITQ				
	Student’s T	–5.857; <0.001	–1.314; 0.207	–0.335; 0.742
	Levene’s L	0.018; 0.896	0.0160; 0.901	<0.001; 0.993
	Shapiro-Wilk’s W	0.980; 0.440	**0.877; 0.004**	**0.877; 0.023**
Treatment - PAI-BOR				
	Student’s T	–0.589; 0.564	–0.726; 0.479	0.892; 0.386
	Levene’s L	0.440; 0.517	0.048; 0.829	0.048; 0.830
	Shapiro-Wilk’s W	0.991; 0.961	0.937; 0.084	0.942; 0.315
Trauma - PAI-BOR				
	Student’s T	**–2.978; 0.009**	–1.416; 0.176	–0.209; 0.836
	Levene’s L	0.417; 0.528	0.031; 0.863	0.557; 0.466
	Shapiro-Wilk’s W	0.986; 0.801	0.943; 0.121	0.941; 0.297
Treatment - PTGI				
	Student’s T	–0.361; 0.723	–0.648; 0.526	–1.699; 0.109
	Levene’s L	<0.001; 0.976	0.530; 0.477	0.543; 0.472
	Shapiro-Wilk’s W	0.961; 0.071	**0.851; 0.001**	**0.889; 0.037**
Trauma - PTGI				
	Student’s T	**–2.938; 0.010**	**–3.966; 0.001**	**–5.673; 0.001**
	Levene’s L	2.160; 0.161	**9.000; 0.008**	3.680; 0.073
	Shapiro-Wilk’s W	0.964; 0.101	0.947; 0.194	0.973; 0.860

Note: Statistic and probability in **bold** when there is a significant difference.

### 3.1 Behavioral Results

#### 3.1.1 Response Accuracy

The behavioral results of the study showed a significant difference in response accuracy between the two participants:

Participant_treatment: The participant who completed the treatment (EMDR) showed a correct response rate of 97.0%, which is equivalent to 582 correct responses. Incorrect responses represented 2.0% (12 responses), and non-responses represented 1.0% (6 responses).

Participant_linebase: In comparison, the baseline participant (who did not complete the treatment) showed a correct response rate of 75.67% (454 correct responses). Incorrect responses represented 4.83% (29 responses), and non-responses were considerably higher, representing 19.50% (117 responses).

#### 3.1.2 Response Times

The average response time also differed markedly between participants, indicating a positive impact of the treatment on the speed of response to stimuli.

Participant_treatment: Response times for correct responses were on average 828.15 ms (standard deviation (SD) = 171.86). For incorrect responses, the average response time was 777.70 ms (SD = 229.1).

Participant_linebase: For the baseline participant, the average response time for correct responses was 1270.66 ms (SD = 324.67), significantly longer than in the treatment group. Response times for incorrect responses were also longer, averaging 1041.75 ms (SD = 339.69).

## 4. Discussion

This RCT case study confirms the initial hypothesis on the efficacy of EMDR for treating PTSD and BPD symptoms. Although the effect of EMDR is comparable to CBT, recent evidence positions it as a promising intervention and alternative to the usual treatments for BPD [53]. These results indicate a moderate effect on traumatic symptoms and a low effect on BPD symptoms and contribute to an emerging body of evidence [18, 19, 20, 53, 54, 55, 56]; however, it is important to consider important differences between these studies and ours. First, a crucial decision was made to differentiate between patients with BPD, with and without PTSD comorbidity, as well as between patients with a clinical diagnosis and those with subclinical symptoms. Second, the effectiveness of EMDR was evaluated in reducing traumatic symptoms, BPD symptoms, or both. Third, the amount, duration, and timing of EMDR application, as well as its combination with other interventions, such as CBT, was included. Additionally, recent studies suggest the importance of including neurobiological markers associated with changes in symptomatology, such as protein levels and methylation related to cortisol and brain-derived neurotrophic factor [19].

As these results support EMDR as a treatment for trauma and PTSD, the reasons behind its efficacy should be explored. The first important factor contributing to its effectiveness may be that EMDR is a psychotherapy that confronts trauma directly, rather than avoiding it. Its objective is for patients to learn to face their trauma through the adaptive processing of traumatic memories. Regardless of the type, EMDR breaks down trauma into its components and organizes them within the therapeutic relationship. This process facilitates the access and processing of these traumatic memories, allowing them to be integrated into the personal history of the individual receiving the treatment. This aspect is particularly critical for BPD, where traumatic experiences can be multiple and intertwined, especially when exposure occurs in early developmental stages, such as childhood [57].

Our findings regarding BPD symptoms, as well as the behavioral effects, reveal descriptive differences in response accuracy and reaction times between the two pilot cases. These single case observations suggest a possible improvement after EMDR but are insufficient to confirm a treatment effect on executive functioning, given the absence of statistical power and control for individual differences. The treated participant exhibited a 97.0% accuracy rate and faster response times, averaging 828.15 ms for correct responses. In contrast, the baseline participant, who did not complete treatment, showed a 75.67% accuracy rate with longer response times, averaging 1270.66 ms. Moreover, the treated participant demonstrated fewer incorrect responses and non-responses. These results suggest that EMDR treatment may enhance both accuracy and processing speed in oddball tasks, reflecting improved executive functions in individuals with trauma symptoms. The reduction in response times and increase in accuracy could be linked to enhanced cognitive efficiency, improved attentional capacity, and an improved post-treatment inhibitory control [54, 58].

However, it is not enough to merely register and organize scattered traumatic memories, as indicated by the EMDR protocol. The bilateral stimulation (e.g., eye movements, auditory stimulation) produced in the desensitization phase of EMDR makes these memories less vivid by competing for available space in working memory. This distances the patient from their memories, enhances interhemispheric communication, promotes relaxation, and produces electroencephalographic activity similar to that observed during REM sleep [59, 60, 61]. Consequently, this facilitates the cortical processing of memories dispersed across different brain areas, which are susceptible to activation by the limbic system (e.g., amygdala) [23, 62, 63, 64]. This reprocessing of traumatic memories reduces symptoms of PTSD, anxiety, and depression that are comorbid with BPD [65]. Additionally, it has moderate to large effects on emotional regulation, interpersonal relationships, and self-concept; three characteristic dimensions of BPD. However, further research is needed to assess its impact on other dimensions of BPD [18, 20].

Additionally, the EMDR protocol has a unique dual advantage. On one hand, it can adapt to diverse patients and dynamic contexts. Given the heterogeneity of patients with BPD, EMDR can be tailored and combined with other interventions in a modular and flexible approach. This compatibility allows for the personalization of treatment based on the specific needs, symptoms, and associated issues of patients with BPD [66]. On the other hand, EMDR possesses a phased structure. It is developed according to a sequence of stages, each aimed at addressing different aspects of trauma and its psychological impact on the recipient [66].

In the first phase, the patient is emotionally and psychologically stabilized through relaxation techniques, psychoeducation, and the development of emotional regulation skills. A sense of safety is created, and the patient is prepared for trauma processing in a safe and controlled environment, preventing further traumatization. The second phase involves direct work with traumatic memories. Techniques such as cognitive restructuring or desensitization are used. This phase seeks to modify the patient’s relationship with their traumatic memories, promoting the integration of these experiences into a coherent and less painful life narrative. The final phase focuses on the reintegration of the individual into their daily life, helping them to develop a new sense of purpose and establish healthy relationships. This phase may also include working on aspects of life affected by trauma, such as self-esteem, confidence, and future planning.

This structure is like other effective treatments, such as CBT. In this regard, therapists and patients organize their sessions based on collaboratively identified problems and objectives. The same results have been reported in BPD, where telehealth benefited patient mental health [54]. This allows the application of techniques from other effective models such as CBT or Dialectical Behavior Therapy (DBT), organizing them according to the complexity of each case [67, 68, 69]. This approach generates a specific treatment that incorporates elements of evidence-based therapies, which explains why the effectiveness of CBT was comparable to that of EMDR.

Based on this therapeutic flexibility and general phased structure, it is not surprising that the teletherapy format works. Meta-analyses have been conclusive in this regard, showing that teletherapy models for PTSD are as effective as face-to-face models in reducing symptoms, as well as in treatment adherence and patient satisfaction [3, 11, 58, 70, 71]. The same results have been reported in BPD, where telehealth benefited patient mental health [72]. Our study contributes to this line of research; however, it must be noted that we did not compare its effect with face-to-face treatment.

Regarding the study’s limitations the asymptomatic arm comprised only five participants, which reduces statistical power, widens confidence intervals, and restricts adjustment for confounders, thereby limiting the generalisability of our findings. Future studies should recruit a larger control group or use matching techniques (e.g., propensity score methods) to minimise this potential bias. Furthermore, the absence of an intention-to-treat analysis presents a limitation worth noting. Given the dropout rate and the number of participants who completed the study, the absence of such an analysis may affect the generalizability of our findings and the interpretation of treatment effects. Future research endeavors should address this limitation by incorporating larger sample sizes and implementing strategies to mitigate attrition bias, thus enabling a more comprehensive evaluation of treatment efficacy and contributing to the advancement of clinical knowledge in this field. A significant limitation to consider was the presence of excessive noise, which hindered EEG analysis. Artifacts generated by muscle movements are a common source of interference that impacts the signal, limiting the ability to conduct a thorough analysis of the collected data [73]. Proper placement of EEG electrodes and the use of appropriate reference electrodes can help mitigate these effects [73]. Finally, follow-up assessments are pending, which will be crucial for understanding the sustainability of treatment effects over time.

## 5. Conclusions

Our study provides evidence for the efficacy of EMDR in reducing PTSD and BPD symptoms, with a moderate effect on traumatic symptoms and a small effect on BPD symptoms. These findings are consistent with previous research that positions EMDR as a promising alternative to treatments such as CBT.

EMDR facilitates the confrontation and adaptive processing of traumatic memories, which is crucial for PTSD and BPD, where traumatic experiences are often multiple and interrelated. Bilateral stimulation in EMDR improves interhemispheric communication and reduces the vividness of traumatic memories, promoting cortical reorganization that reduces symptoms of PTSD and BPD. In addition, the structure of EMDR allows for personalization of treatment by incorporating techniques from other therapeutic models such as CBT and DBT.

Despite the findings, elements that can be considered limitations of the studies, such as comorbidity between PTSD and BPD, as well as symptom severity and the combination of EMDR with other therapies, need to be addressed. To this end, it is also important that future studies address the inclusion of neurobiological markers to deepen the understanding of symptomatology.

In conclusion, EMDR has been shown to be an effective and adaptive intervention, so it is necessary to advance the development of further research in this area.

## Availability of Data and Materials

Data generated and analyzed during this study are available from the corresponding author on request.

## Supplementary Material

Supplementary material associated with this article can be found, in the online version, at https://doi.org/10.31083/AP40031.

## References

[1] Hoogsteder LM, Ten Thije L, Schippers EE, Stams GJJM (2022). A Meta-Analysis of the Effectiveness of EMDR and TF-CBT in Reducing Trauma Symptoms and Externalizing Behavior Problems in Adolescents. *International Journal of Offender Therapy and Comparative Criminology*.

[2] Jongh AD, Broeke ET, Farrell D, Maxfield L (2020). Empirically Supported Psychological Treatments: EMDR Therapy. *The Oxford Handbook of Traumatic Stress Disorders*.

[3] McClellan MJ, Osbaldiston R, Wu R, Yeager R, Monroe AD, McQueen T (2022). The effectiveness of telepsychology with veterans: A meta-analysis of services delivered by videoconference and phone. *Psychological Services*.

[4] Roepke AM (2015). Psychosocial interventions and posttraumatic growth: a meta-analysis. *Journal of Consulting and Clinical Psychology*.

[5] Seidler GH, Wagner FE (2006). Comparing the efficacy of EMDR and trauma-focused cognitive-behavioral therapy in the treatment of PTSD: a meta-analytic study. *Psychological Medicine*.

[6] APA-American Psychiatric Association (2013). *Diagnostic and Statistical Manual of Mental Disorders. Fifth Edition (DSM-5)*.

[7] World Health Organization (2013). WHO guidelines on conditions specifically related to stress. https://www.who.int/publications/i/item/9789241505406.

[8] Rasines-Laudes P, Serrano-Pintado I (2023). Efficacy of EMDR in Post-Traumatic Stress Disorder: A Systematic Review and Meta-analysis of Randomized Clinical Trials. *Psicothema*.

[9] Khan AM, Dar S, Ahmed R, Bachu R, Adnan M, Kotapati VP (2018). Cognitive Behavioral Therapy versus Eye Movement Desensitization and Reprocessing in Patients with Post-traumatic Stress Disorder: Systematic Review and Meta-analysis of Randomized Clinical Trials. *Cureus*.

[10] Rodenburg R, Benjamin A, de Roos C, Meijer AM, Stams GJ (2009). Efficacy of EMDR in children: a meta-analysis. *Clinical Psychology Review*.

[11] Shaker AA, Austin SF, Storebø OJ, Schaug JP, Ayad A, Sørensen JA (2023). Psychiatric Treatment Conducted via Telemedicine Versus In-Person Modality in Posttraumatic Stress Disorder, Mood Disorders, and Anxiety Disorders: Systematic Review and Meta-Analysis. *JMIR Mental Health*.

[12] Valiente-Gómez A, Moreno-Alcázar A, Treen D, Cedrón C, Colom F, Pérez V (2017). EMDR beyond PTSD: A Systematic Literature Review. *Frontiers in Psychology*.

[13] Perlini C, Donisi V, Rossetti MG, Moltrasio C, Bellani M, Brambilla P (2020). The potential role of EMDR on trauma in affective disorders: A narrative review. *Journal of Affective Disorders*.

[14] Mosquera D, Leeds AM, Gonzalez A (2016). Application of EMDR therapy for borderline personality disorder. *Journal of EMDR Practice and Research*.

[15] Martín-Blanco A, Soler J, Villalta L, Feliu-Soler A, Elices M, Pérez V (2014). Exploring the interaction between childhood maltreatment and temperamental traits on the severity of borderline personality disorder. *Comprehensive Psychiatry*.

[16] Rosada C, Bauer M, Golde S, Metz S, Roepke S, Otte C (2023). Childhood trauma and cortical thickness in healthy women, women with post-traumatic stress disorder, and women with borderline personality disorder. *Psychoneuroendocrinology*.

[17] Cloitre M, Garvert DW, Brewin CR, Bryant RA, Maercker A (2013). Evidence for proposed ICD-11 PTSD and complex PTSD: a latent profile analysis. *European Journal of Psychotraumatology*.

[18] Hafkemeijer L, Slotema K, de Haard N, de Jongh A (2023). Case report: Brief, intensive EMDR therapy for borderline personality disorder: results of two case studies with one year follow-up. *Frontiers in Psychiatry*.

[19] Snoek A, Beekman ATF, Dekker J, Aarts I, van Grootheest G, Blankers M (2020). A randomized controlled trial comparing the clinical efficacy and cost-effectiveness of eye movement desensitization and reprocessing (EMDR) and integrated EMDR-Dialectical Behavioural Therapy (DBT) in the treatment of patients with post-traumatic stress disorder and comorbid (Sub)clinical borderline personality disorder: study design. *BMC Psychiatry*.

[20] Brown S, Shapiro F (2006). EMDR in the treatment of borderline personality disorder. *Clinical Case Studies*.

[21] Leiva-Bianchi M, Ahumada F, Araneda A, Botella J (2018). What is the Psychosocial Impact of Disasters? A Meta-Analysis. *Issues in Mental Health Nursing*.

[22] Farina B, Imperatori C, Quintiliani MI, Castelli Gattinara P, Onofri A, Lepore M (2015). Neurophysiological correlates of eye movement desensitization and reprocessing sessions: preliminary evidence for traumatic memories integration. *Clinical Physiology and Functional Imaging*.

[23] Pagani M, Högberg G, Fernandez I, Siracusano A (2013). Correlates of EMDR therapy in functional and structural neuroimaging: A critical summary of recent findings. *Journal of EMDR Practice and Research*.

[24] Balkin RS, Lenz AS, Russo GM, Powell BW, Gregory HM (2022). Effectiveness of EMDR for decreasing symptoms of over-arousal: A meta-analysis. *Journal of Counseling and Development*.

[25] Askovic M, Soh N, Elhindi J, Harris AWF (2023). Neurofeedback for post-traumatic stress disorder: systematic review and meta-analysis of clinical and neurophysiological outcomes. *European Journal of Psychotraumatology*.

[26] Babaskina L, Afanasyeva N, Semyonkina M, Myasnyankina O, Sushko N (2023). Effectiveness of Neurofeedback Training for Patients with Personality Disorders: A Systematic Review. *Iranian Journal of Psychiatry*.

[27] Choi YJ, Choi EJ, Ko E (2023). Neurofeedback Effect on Symptoms of Posttraumatic Stress Disorder: A Systematic Review and Meta-Analysis. *Applied Psychophysiology and Biofeedback*.

[28] Steingrimsson S, Bilonic G, Ekelund AC, Larson T, Stadig I, Svensson M (2020). Electroencephalography-based neurofeedback as treatment for post-traumatic stress disorder: A systematic review and meta-analysis. *European Psychiatry: the Journal of the Association of European Psychiatrists*.

[29] Sağaltıcı E, Çetinkaya M, Kocamer Şahin Ş, Gülen B, Karaman Ş (2022). Recent Traumatic Episode Protocol EMDR Applied Online for COVID-19-Related Symptoms of Turkish Health Care Workers Diagnosed with COVID-19-Related PTSD: A Pilot Study. *Alpha Psychiatry*.

[30] Faul F, Erdfelder E, Lang AG, Buchner A (2007). G*Power 3: a flexible statistical power analysis program for the social, behavioral, and biomedical sciences. *Behavior Research Methods*.

[31] Faul F, Erdfelder E, Buchner A, Lang AG (2009). Statistical power analyses using G*Power 3.1: tests for correlation and regression analyses. *Behavior Research Methods*.

[32] Boselli M, Parrino L, Smerieri A, Terzano MG (1998). Effect of age on EEG arousals in normal sleep. *Sleep*.

[33] Venegas B, Avila F, Aylwin M (2021). Ethics Committee, University of Talca, Chile: Approval Act for FONDECYT Project No. 1190578. https://cec.utalca.cl/.

[34] Cloitre M (2020). ICD-11 complex post-traumatic stress disorder: simplifying diagnosis in trauma populations. *The British Journal of Psychiatry: the Journal of Mental Science*.

[35] Cloitre M, Hyland P, Prins A, Shevlin M (2021). The international trauma questionnaire (ITQ) measures reliable and clinically significant treatment-related change in PTSD and complex PTSD. *European Journal of Psychotraumatology*.

[36] Cloitre M, Shevlin M, Brewin CR, Bisson JI, Roberts NP, Maercker A (2018). The International Trauma Questionnaire: development of a self-report measure of ICD-11 PTSD and complex PTSD. *Acta Psychiatrica Scandinavica*.

[37] Fresno A, Ramos Alvarado N, Núñez D, Ulloa JL, Arriagada J, Cloitre M (2023). Initial validation of the International Trauma Questionnaire (ITQ) in a sample of Chilean adults. *European Journal of Psychotraumatology*.

[38] Distel MA, De Moor MHM, Boomsma DI (2009). Nederlandse vertaling van de Personality Assessment Inventory-Borderline kenmerken schaal (PAI-BOR): normgegevens, factorstructuur en betrouwbaarheid. *Psychologie en Gezondheid*.

[39] Jackson KM, Trull TJ (2001). The factor structure of the Personality Assessment Inventory-Borderline Features (PAI-BOR) Scale in a nonclinical sample. *Journal of Personality Disorders*.

[40] De Moor MHM, Distel MA, Trull TJ, Boomsma DI (2009). Assessment of borderline personality features in population samples: is the Personality Assessment Inventory-Borderline Features scale measurement invariant across sex and age?. *Psychological Assessment*.

[41] Leiva-Bianchi MC, Araneda AC (2013). Validation of the Davidson Trauma Scale in its original and a new shorter version in people exposed to the F-27 earthquake in Chile. *European Journal of Psychotraumatology*.

[42] The jamovi project (2024). jamovi (Version 2.2.5). *Computer Software*.

[43] The MathWorks Inc (2022). MATLAB version: 9.13.0 (R2022b). *Computer Software*.

[44] Huynh H (1978). Some approximate tests for repeated measurement designs. *Psychometrika*.

[45] Leiva-Bianchi M, Cornejo F, Fresno A, Rojas C, Serrano C (2018). Effectiveness of cognitive-behavioural therapy for post-disaster distress in post-traumatic stress symptoms after Chilean earthquake and tsunami. *Gaceta Sanitaria*.

[46] Leiva-Bianchi M, Pardo A (2012). *Cómo Escoger Estrategias Robustas Para Valorar Medidas Reptidas?*.

[47] Blanca MJ, Arnau J, López-Montiel D, Bono R, Bendayan R (2013). Skewness and kurtosis in real data samples. *Methodology*.

[48] Ferguson CJ (2009). An Effect Size Primer: A Guide for Clinicians and Researchers. *Professional Psychology: Research and Practice*.

[49] Bakeman R (2005). Recommended effect size statistics for repeated measures designs. *Behavior Research Methods*.

[50] Olejnik S, Algina J (2003). Generalized eta and omega squared statistics: measures of effect size for some common research designs. *Psychological Methods*.

[51] Mili R, Bouaziz B, Maalel A, Gargouri F (2023). EEG and fMRI Artifact Detection Techniques: A Survey of Recent Developments. *SN Computer Science*.

[52] Jiang X, Bian GB, Tian Z (2019). Removal of Artifacts from EEG Signals: A Review. *Sensors (Basel, Switzerland)*.

[53] Scelles C, Bulnes LC (2021). EMDR as Treatment Option for Conditions Other Than PTSD: A Systematic Review. *Frontiers in Psychology*.

[54] Hudays A, Gallagher R, Hazazi A, Arishi A, Bahari G (2022). Eye Movement Desensitization and Reprocessing versus Cognitive Behavior Therapy for Treating Post-Traumatic Stress Disorder: A Systematic Review and Meta-Analysis. *International Journal of Environmental Research and Public Health*.

[55] Momeni Safarabad N, Asgharnejad Farid AA, Gharraee B, Habibi M (2018). Treatment of a Patient with Borderline Personality Disorder Based on Phase-Oriented Model of Eye Movement Desensitization and Reprocessing (EMDR): A Case Report. *Iranian Journal of Psychiatry*.

[56] Wilhelmus B, Marissen MAE, van den Berg D, Driessen A, Deen ML, Slotema K (2023). Adding EMDR for PTSD at the onset of treatment of borderline personality disorder: A pilot study. *Journal of Behavior Therapy and Experimental Psychiatry*.

[57] Porter C, Palmier-Claus J, Branitsky A, Mansell W, Warwick H, Varese F (2020). Childhood adversity and borderline personality disorder: a meta-analysis. *Acta Psychiatrica Scandinavica*.

[58] Kelber MS, Smolenski DJ, Boyd C, Shank LM, Bellanti DM, Milligan T (2024). Evidence-based telehealth interventions for posttraumatic stress disorder, depression, and anxiety: A systematic review and meta-analysis. *Journal of Telemedicine and Telecare*.

[59] Gunter RW, Bodner GE (2009). But How? Recent Progress in the Search for Treatment Mechanisms. *Journal of EMDR Practice and Research*.

[60] Landin-Romero R, Moreno-Alcazar A, Pagani M, Amann BL (2018). How Does Eye Movement Desensitization and Reprocessing Therapy Work? A Systematic Review on Suggested Mechanisms of Action. *Frontiers in Psychology*.

[61] Wadji DL, Martin-Soelch C, Camos V (2022). Can working memory account for EMDR efficacy in PTSD?. *BMC Psychology*.

[62] Pagani M, Di Lorenzo G, Monaco L, Daverio A, Giannoudas I, La Porta P (2015). Neurobiological response to EMDR therapy in clients with different psychological traumas. *Frontiers in Psychology*.

[63] Pagani M, Di Lorenzo G, Verardo AR, Nicolais G, Monaco L, Lauretti G (2012). Neurobiological correlates of EMDR monitoring - an EEG study. *PloS One*.

[64] Rousseau PF, El Khoury-Malhame M, Reynaud E, Zendjidjian X, Samuelian JC, Khalfa S (2019). Neurobiological correlates of EMDR therapy effect in PTSD. *European Journal of Trauma & Dissociation*.

[65] Chen R, Gillespie A, Zhao Y, Xi Y, Ren Y, McLean L (2018). The Efficacy of Eye Movement Desensitization and Reprocessing in Children and Adults Who Have Experienced Complex Childhood Trauma: A Systematic Review of Randomized Controlled Trials. *Frontiers in Psychology*.

[66] Karatzias T, Murphy P, Cloitre M, Bisson J, Roberts N, Shevlin M (2019). Psychological interventions for ICD-11 complex PTSD symptoms: systematic review and meta-analysis. *Psychological Medicine*.

[67] Neacsiu AD, Rizvi SL, Linehan MM (2010). Dialectical behavior therapy skills use as a mediator and outcome of treatment for borderline personality disorder. *Behaviour Research and Therapy*.

[68] Soler J, Pascual JC, Tiana T, Cebrià A, Barrachina J, Campins MJ (2009). Dialectical behaviour therapy skills training compared to standard group therapy in borderline personality disorder: a 3-month randomised controlled clinical trial. *Behaviour Research and Therapy*.

[69] Verheul R, Van Den Bosch LMC, Koeter MWJ, De Ridder MAJ, Stijnen T, Van Den Brink W (2003). Dialectical behaviour therapy for women with borderline personality disorder: 12-month, randomised clinical trial in The Netherlands. *The British Journal of Psychiatry: the Journal of Mental Science*.

[70] Olthuis JV, Wozney L, Asmundson GJG, Cramm H, Lingley-Pottie P, McGrath PJ (2016). Distance-delivered interventions for PTSD: A systematic review and meta-analysis. *Journal of Anxiety Disorders*.

[71] Scott AM, Bakhit M, Greenwood H, Cardona M, Clark J, Krzyzaniak N (2022). Real-Time Telehealth Versus Face-to-Face Management for Patients with PTSD in Primary Care: A Systematic Review and Meta-Analysis. *The Journal of Clinical Psychiatry*.

[72] Heidari P, Broadbear JH, Brown R, Dharwadkar NP, Rao S (2023). Mental health support for and telehealth use by Australians living with borderline personality disorder during the onset of the COVID-19 pandemic: A national study. *Digital Health*.

[73] Luck S (2014). *An Introduction to the Event-Related Potential Technique*.

